# MicroRNA-486-5p Suppresses Lung Cancer *via* Downregulating mTOR Signaling *In Vitro* and *In Vivo*

**DOI:** 10.3389/fonc.2021.655236

**Published:** 2021-05-20

**Authors:** Lei Ding, Wu Tian, Hui Zhang, Wanqiu Li, Chunyu Ji, Yuanyuan Wang, Yanli Li

**Affiliations:** ^1^ Lab for Noncoding RNA & Cancer, School of Life Sciences, Shanghai University, Shanghai, China; ^2^ Department of Pancreatic Surgery, Shanghai Cancer Centre, Fudan University, Shanghai, China; ^3^ Department of General Surgery, Orthopedics Hospital of Guizhou Province, Guiyang, China; ^4^ Department of Thoracic Surgery, Shanghai Chest Hospital, Jiaotong University Medical School, Shanghai, China; ^5^ Department of Respiratory and Critical Care Medicine, East Hospital, Tongji University School of Medicine, Shanghai, China

**Keywords:** NSCLC, miR-486-5p, mTOR signaling, RSK, p70S6K

## Abstract

Lung cancer is one of the central causes of tumor-related deaths globally, of which non-small cell lung cancer (NSCLC) takes up about 85%. As key regulators of various biological processes, microRNAs (miRNAs) have been verified as crucial factors in NSCLC. To elucidate the role of miR-486-5p in the mTOR pathway, we investigated its role in NSCLC and related signaling. Our results confirmed that miR-486-5p was downregulated in most of human NSCLC tissue samples and cell lines. Further study confirmed that it inhibited NSCLC through repression of the mTOR pathway *via* targeting both ribosomal proteins S6 kinase A1 (RPS6KA1, RSK) and ribosomal proteins S6 kinase B1 (RPS6KB1, p70S6K), which are critical components of the mTOR signaling. Additionally, miR-486-5p impeded tumor growth *in vivo* and inhibited tumor metastasis through repression of the epithelial-mesenchymal transition (EMT). Taken together, our study verified the role that miR-486-5p exerts in NSCLC, and its expression pattern in the different stages and morphologies of NSCLC makes it a promising biomarker in the early diagnosis of the disease.

## Introduction

Lung cancer is known for its high occurrence and mortality rate both domestically and abroad (1), in which non-small cell lung cancer (NSCLC) accounts for about 85%. As a result, the prognosis of the disease remains poor (2, 3). Further categorization of it comes to lung adenocarcinoma (LUAD, 40–50% of all cases), lung squamous cell carcinoma (LUSC, 20–30% of all cases) and large cell lung cancer.

microRNAs (miRNAs) are noncoding RNAs with single strand of ~22 nucleotides by which about 60% of genes are regulated post-transcriptionally through either translational repression or mRNA degradation (4). MiRNAs are reported to play critical roles in diverse biological processes including cell proliferation (5, 6), cell cycle (7–9), cell migration (10–12), cell apoptosis (13, 14) immune response (15) and tumorigenesis (16, 17), suggesting their crucial roles in the development of various malignancies. To date, numerous miRNAs have been studied in NSCLC, revealing their crucial roles in the tumorigenesis and development of NSCLC (18–21). shedding some bright light in the diagnosis and treatment of lung cancer.

Our previous work has demonstrated the downregulation of miR-486-5p in NSCLC cell lines and patients’ tissue samples and it suppressed NSCLC cell growth and promoted apoptosis by direct repression of cyclin-dependent kinase 4 (CDK4) (22). However, it was also reported to play an oncogenic role by targeting tensin homolog deleted on chromosome ten (PTEN) and subsequently activating the phosphatidylinositol 3-kinase (PI3K)/AKT signaling (23). Here, we identified targets of miRNA-586-5p, RSK and p70S6K, which are in regulating mTOR signaling. In short, our study showed that miR-486-5p significantly inhibited the mTOR pathway through targeting RSK and p70S6K, resulting in suppressive effects in NSCLC.

## Materials and Methods

### Specimen Collection and Ethical Statement

68 NSCLC tissue specimens and their paired noncancerous tissues were collected from Shanghai Chest Hospital (Shanghai, China) with informed consent obtained. Approval of the experiments in this study by the Ethics Committee of Shanghai Chest Hospital was obtained. All patients’ information used in this study is listed in **Table S1**.

### Cell Culture

Five human NSCLC cell lines (95-D, A549, H1975, HCC827, and PC-9), cells from the normal human bronchial epithelium (HBE), and HEK293T were purchased from the Cell Bank of the Chinese Academy of Sciences (Shanghai, China). H1299 cells were bought from the American Type Culture Collection (ATCC, Manassas, VA, USA). Authentication of all the cell lines were with the short tandem repeat (STR) method was performed.

RPMI-1640 medium (Gibco, Gaithersburg, MD, USA) was used for 95-D, H1299, H1975, HCC827, and PC-9 culturing and DMEM (Gibco, Gaithersburg, MD, USA) for A549, HBE, and HEK293T at 37°C plus an atmosphere of 5% CO2. 10% fetal bovine serum (FBS, HyClone Laboratories, Logan, UT, USA) and an antibiotic cocktail (100 U/ml penicillin and 100 μg/ml streptomycin) (Gibco, Gaithersburg, MD, USA) were added into all media for cell culture.

### Construction of Plasmid, siRNA, and Cell Transfection

Lentivirus construction and infection were performed as formerly introduced (24). In brief, after digestion with *Eco*R I and *Xba* I (Takara, Dalian, China), pre-miR-486-5p sequence was cloned into pLenti vector (Invitrogen, Carlsbad, CA, USA) (named pLenti-miR-486). Viral particles containing pLenti or pLenti-miR-486 were collected to infect A549 and H1299 cells and further sorted with flow cytometry.

The wild-type (WT) RSK and p70S6K 3’UTR fragments and their corresponding mutant (Mut) fragments were cloned into pGL3-basic (pGL3, Promega, Madison, WI, USA) luciferase reporter vector to confirm their direct binding with miR-486-5p. These pGL3-derived vectors and miR-486-5p mimics were then cotransfected into 293T cells, and the Dual-Luciferase Reporter Assay (Promega, Madison, WI, USA) was applied for luciferase activity measurement as previously described (25).

The sequences of RSK and p70S6K were constructed into the pcDNA3.1 plasmid. siRNAs for RSK (siRSK #1-3) and p70S6K (sip70S6K #1-3) were synthesized by RIBOBIO (Guangzhou, China). All siRNA sequences were listed in **Table S2**. Lipofectamine 2000 reagent (Invitrogen, Carlsbad, CA, USA) was used for transient transfection. All the constructed vectors were sequenced (Sangon Biotech, Shanghai, China) and the primers are listed in **Table S3**.

### qRT-PCR and Western Blot

Total RNA was extracted, and reverse transfected according to manufactures’ guide. The RNA level was quantified by qRT-PCR using an SYBR Green PCR master mix (TaKaRa, Dalian, China). The endogenous controls for mRNA and miRNA were 18S RNA and U6 snRNA, respectively. The relative quantification (2^-ΔΔCT^) method was used for results analyzing.

Total protein was extracted from the cells using RIPA lysis buffer (CWBIO, Beijing, China) and quantified with a Bradford Kit (Bio-Rad, Hercules, California, USA). Sodium dodecyl sulfate-polyacrylamide gel electrophoresis (SDS-PAGE) was applied to separate proteins, which were then transferred to a polyvinylidene difluoride (PVDF) membrane (Millipore Corporation, Billerica, MA, USA). After blocking with non-fat powdered milk at room temperature for 1 h following an incubation with rabbit anti-RSK, anti-p70S6K, anti-p-p70S6K, anti-mTOR, anti-p-mTOR, anti-E-Cad, and anti-N-Cad antibodies overnight at 4°C. After incubation with a goat-anti-rabbit secondary antibody conjugated to horseradish peroxidase (HRP) (1:10000, Transgene Biotech, Beijing, China), protein bands were photographed with a chemiluminescent HRP substrate (Millipore, Billerica, MA, USA) and imaged with an E-Gel Imager (Biotanon, Shanghai, China). All experiments were repeated for three times and the representative images of the protein bands are shown in the figures. The number indicates the grayscale value of the protein bands relative to GAPDH. All the antibodies used in this study are listed in **Table S4**.

### CCK-8 and Colony Formation Assay

CCK-8 (Dojindo, Japan) and colony formation assays were performed according to a formerly described protocol (24). For CCK-8 assay, 2500 cells per well were seeded in 96-well plates and incubated with 5% CCK-8 for 2.5 h every 24 h, and then the absorbance of the supernatant at 450 nm was measured with a plate reader. For colony formation assay, 500 cells were seeded in 6-well plates for 12-14 d until the macroscopic colonies form, followed by crystal violet staining and photographing.

### Flow Cytometry Assay

Flow cytometry (BD Biosciences, San Jose, CA, USA) was used for cell sorting, cell cycle analysis apoptosis detection. For apoptotic rate assessment, pLenti/pLenti-miR-486 stably transfected cells were treated with actinomycin D (AMD, 5 μg/mL) for 4 h and then stained for Annexin-V APC/7AAD (Keygentec, Jiangsu, China) prior to analysis by flow cytometry. Transient transfected cells were stained for Annexin-V FITC/PI (BD Biosciences, San Jose, CA, USA) following treatment with actinomycin D and analyzed by flow cytometry.

### Wound Healing Assay

Cells were scratched with 20 μL tips and washed with PBS twice, and the position was recorded by using a 10× lens on a light microscope and a digital camera. After 24 h (H1299) or 48 h (A549), cell positions were recorded again.

### Mouse Xenograft Model

Six-week-old female SCID mice were purchased from the Shanghai Laboratory Animal Center (SLAC, Shanghai, China) and maintained under specific-pathogen-free (SPF) conditions with a healthy state test by SLAC. After random assignment to one of 2 groups, with 5 mice in each group, each mouse was injected subcutaneously in the right flank with 5 × 10^6^ H1299 cells and intravenously with 2.5 × 10^6^ cells transfected with pLenti or pLenti-miR-486. Tumor diameters were measured with calipers weekly, and the formula: volume = length × width^2^/2 was applied to determine the tumor size. Eight weeks after the injection, the mice were sacrificed with CO_2_ without using any anesthesia, with the xenografts excised and weighed. The results were analyzed between the groups. All experimental protocols were approved under the institutional Animal Care and Use Committee of Shanghai University (Shanghai, China).

### IHC Analysis

The xenografts were embedded in paraffin, then deparaffinized and rehydrated, followed by antigen retrieval. The sections were incubated with primary antibodies against Ki67, RSK, p70S6K, p-p70S6K, mTOR, p-mTOR, E-cad, and N-cad followed by incubation with secondary antibodies. The sections were then stained with 3, 3′-diaminobenzidine reaction solution and photographed using a digitalized microscope camera. All the antibodies used in this study are listed in **Table S4**.

### Bioinformatics

miRWalk, together with miRTarBase and TargetScan, was used for targets prediction. UALCAN and Kaplan-Meier Plotter were used to analyze the data from TCGA. All the databases are listed in **Table S5**.

### Statistical Analysis

Data were analyzed with GraphPad Prism software 8.0 (GraphPad Software, San Diego, CA, USA) and IBM SPSS v25.0 software (Abbott Laboratories, Chicago, IL, USA). Results were presented as the mean ± SD. Statistical analyses were performed using the Student’s t-test and one-way ANOVA. Pearson’s chi-square test was used to analyze the associations between miR-486-5p and RSK and p70S6K expression in tissue samples. All the experiments were repeated at least three times independently. Statistical significance was established at p < 0.05.

## Results

### miR-486-5p Is Downregulated in NSCLC

**Figure 1:**
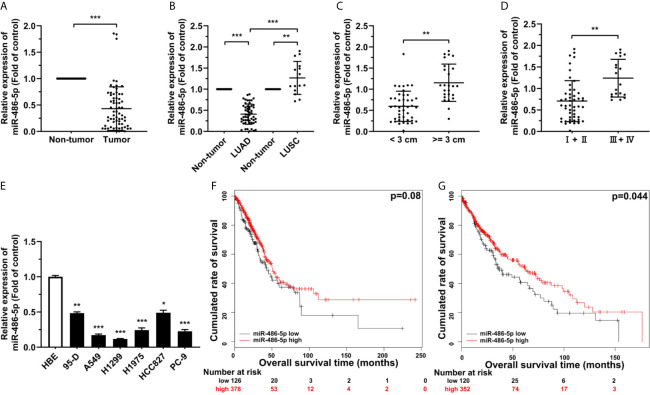
miR-486-5p is downregulated in NSCLC and correlated with patients’ longer survival. **(A)** The expression of miR-486-5p in 68 pairs of NSCLC specimens relative to their adjacent normal tissues. ***p < 0.001. **(B)** The miR-486-5p expression in the LUAD (N=53) and LUSC (N=15) tissue samples. **p < 0.01; ***p < 0.001. **(C)** miR-486-5p expression in tissue samples with diameters < 3 cm (N=43) and >= 3 cm (N=25). **p < 0.01. **(D)** miR-486-5p expression in TNM stage I and stage II (N=49) NSCLC tissues and stage III and stage IV (N=19) tissues. **p < 0.01. **(E)** The expression of miR-486-5p in NSCLC cell lines 95-D, A549, H1299, H1975, HCC827 and PC-9. HBE cells were used as normal control. *p < 0.05; **p < 0.01; ***p < 0.001. **(F, G)** Retrospective analysis of Kaplan-Meier plots for miR-486-5p expression in association with overall survival of LUAD **(F)** and LUSC **(G)** patients.

To study the relationship between the expression of miR-486-5p and NSCLC, quantitative real-time polymerase chain reaction (qRT-PCR) results showed the marked downregulation of miR-486-5p in the 68 human NSCLC samples than in their paired adjacent non-tumor tissues (**Figure 1A**), the expression of miR-486-5p based on the pathological type, namely LUAD and LUSC samples, the tumor size, and the TNM neoplasm staging were also compared. It turned out that miR-486-5p was mainly downregulated in LUAD (**Figure 1B**) and samples with diameters <3 cm (**Figure 1C**) and the stage I and II samples (**Figure 1D**), revealing that miR-486-5p might play different roles in the tumorigenesis of LUAD and LUSC, and is positively regulated in advanced NSCLC.

miR-486-5p was also downregulated in most of our NSCLC cell lines, comparing to the non-transformed human lung epithelial cell line HBE (**Figure 1E**). A549 and H1299 cell lines were chosen for further study, for miR-486-5p expression in these two cell lines was relatively the lowest. Moreover, analyzing The Cancer Genome Atlas (TCGA) database also confirmed the downregulation of miR-486-5p in both LUAD and LUSC tissues (**Figure S1**) (26), and we can also conclude from TCGA database that higher miR-486-5p level is positively correlated with higher probability of survival both in LUAD (**Figure 1F**) and LUSC (**Figure 1G**) patients (27). To sum up, our results indicated that miR-486-5p might function as cancer suppressor in NSCLC.

### miR-486-5p Inhibits NSCLC Proliferation

**Figure 2:**
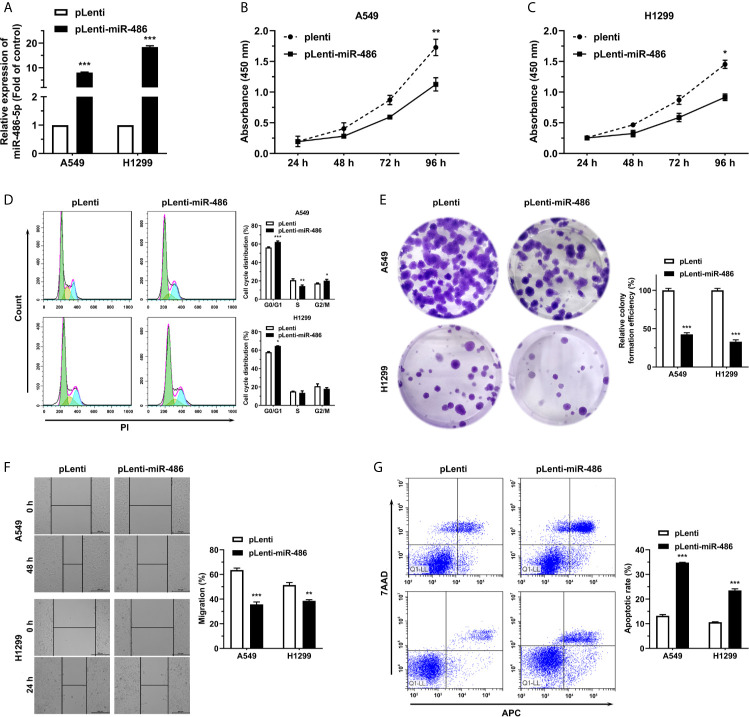
miR-486-5p suppresses the proliferation and migration of NSCLC cells and promotes cell apoptosis. **(A)** The expression of miR-486-5p in pLenti/pLenti-miR-486 stably transfected A549 and H1299 cells. ***p < 0.001. **(B–G)** The effect of miR-486-5p on the proliferation **(B, C)**, cell cycle progress **(D)**, colony formation **(E)**, migration **(F)** and apoptosis **(G)** of A549 and H1299 cells stably transfected with pLenti or pLenti-miR-486. *p < 0.05; **p < 0.01; ***p < 0.001.

miR-486-5p (pLenti-miR-486) or an empty vector (pLenti) was forced expressed with lentivirus to assess the roles of miR-486-5p on NSCLC cell phenotypes. The successful transfection of the vectors can be indicated from the fluorescence intensity of green fluorescence protein (GFP) (**Figure S2**). miR-486-5p expression was upregulated remarkably in the pLenti-miR-486-expressing A4549 and H1299 cells relative to the pLenti-expressing ones, with nearly 8- and 18-fold increases respectively (**Figure 2A**). CCK-8 assay revealed that miR-486-5p overexpression inhibited H1299 and A549 cell proliferation (**Figures 2B, C**). In search of the underlying mechanism of the suppressing role that miR-486-5p played in NSCLC, we analyzed its function on cell cycle, colony formation, cell migration and cell apoptosis. It turned out that the cell cycle progress of A549 and H1299 was retarded by miR-486-5p (**Figure 2D**), as well as the colony formation (**Figure 2E**) and migration rate (**Figure 2F**), with the latter determined by wound healing assay. On the contrary, the cell apoptosis was considerably facilitated by miR-486-5p (**Figure 2G**). In conclusion, these results verified that miR-486-5p acted as a suppressor in the NSCLC.

### RSK and p70S6K Are Targets of miR-486-5p

**Figure 3:**
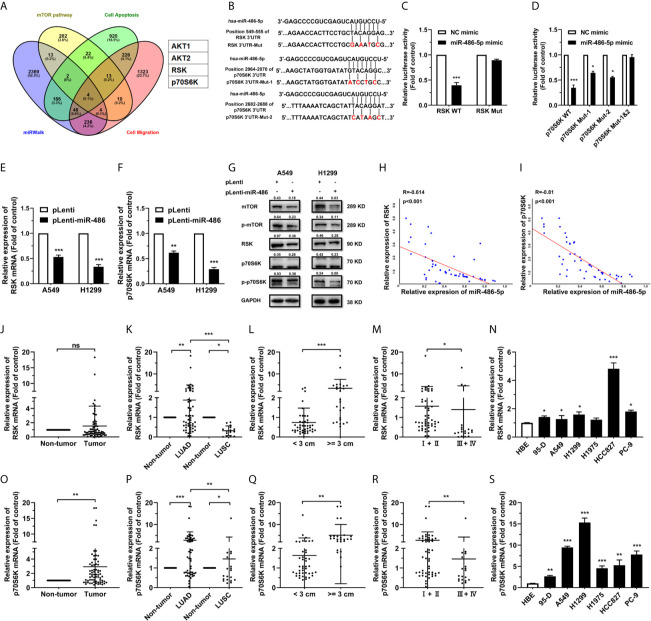
miR-486-5p directly targets RSK and p70S6K. **(A)** The predicted targets of miR-486-5p by miRWalk 2.0. AKT1, AKT2, RSK and p70S6K stand out by restricting the results in the mTOR pathway, cell-apoptosis-associated proteins and cell-migration-associated proteins. **(B)** Schematic representation of the binding sites of miR-486-5p and the 3’UTRs of RSK and p70S6K. **(C, D)** The luciferase activity in HEK293 cells cotransfected with, and RSK or p70S6K 3’UTR, pRL vector (RSK/p70S6K WT), or with NC or miR-486-5p mimic, and the RSK/p70S6K 3’mUTR, and pRL vector (RSK/p70S6K Mut). *p < 0.05; ***p < 0.001. **(E–G)** The mRNA **(E, F)** and protein **(G)** expression of RSK and p70S6K in pLenti/pLenti-miR-486 transfected A549 and H1299 cells. **p < 0.01; ***p < 0.001. **(H, I)** Correlation between miR-486-5p and RSK/p70S6K RNA expression in human LUAD tissue samples. Two-tailed Spearman’s correlation analysis. R, coefficient of association; P, *p* value. **(J–M)** The RNA expression of RSK in human NSCLC tissue samples (N=68). ns, non-significant. The expression between the LUAD (N=53) and LUSC (N=15) specimens. **(K)**, tissues with diameters < 3 cm (N=43) or >=3 cm (N=25). **(L)**, and TNM stage I/II (N=49) and stage III/IV (N=19). **(M)** samples was compared. *p < 0.05; **p < 0.01; ***p < 0.001. **(N)** The RNA expression of RSK in human NSCLC cell lines 95-D, A549, H1299, H1975, HCC827 and PC-9. HBE cells were used as normal control. *p < 0.05; ***p < 0.001. **(O, R)** The RNA expression of p70S6K in human NSCLC tissue samples (N=68). The expression between the LUAD (N=53) and LUSC (N=15) specimens. **(P)**, tissues with diameters < 3 cm (N=43) or >=3 cm (N=25). **(Q)**, and TNM stage I/II (N=49) and stage III/IV (N=19). **(R)** samples was compared. *p < 0.05; **p < 0.01; ***p < 0.001. **(S)** The expression of p70S6K RNA in human NSCLC cell lines 95-D, A549, H1299, H1975, HCC827 and PC-9. HBE cells were used as normal control. *p < 0.05; **p < 0.01; ***p < 0.001.

To elucidate the mechanism by which miR-486-5p hinders NSCLC progress, we predicted its potential targets using miRWalk 3.0 (28). Four candidates stood out after overlapping the predicted targets by miRWalk with the mTOR-, apoptosis- and cell migration-related proteins (**Figure 3A**). Given the functions of miR-486-5p in NSCLC cell lines, we ultimately chose the target genes RSK and p70S6K. The wild type and mutated binding sites for miR-486-5p with RSK and p70S6K are demonstrated in **Figure 3B**, and their binding relationship was determined by dual-luciferase reporter assay. Significant decrease of the relative luciferase activity in HEK293T cells cotransfected with the 3’ untranslated region (3’UTR) of RSK and p70S6K (pGL3-RSK/pGL3-p70S6K WT 3’UTR), pRL vector, and miR-486-5p mimic compared to the control (cotransfected with RSK and p70S6K 3’UTR, pRL vector and NC mimic) was observed, while no significant change in luciferase activity was found in cotransfection of miR-486-5p mimic with the RSK and p70S6K 3’mUTR (pGL3-RSK/pGL3-p70S6K Mut 3’UTR), indicating that miR-486-5p has direct binding sites in RSK and p70S6K mRNA 3’UTRs (**Figures 3C, D**).

To determine how miR-486-5p affected the expression of RSK and p70S6K, we investigated the expression of RSK and p70S6K in pLenti-miR-486 A549 and H1299 cells compared to that in pLenti cells by qRT-PCR and western blot. RSK and p70S6K expression was decreased by miR-486-5p on both the mRNA (**Figures 3E, F**) and protein (**Figure 3G**) levels in A549 and H1299 cells. Also, RSK and p70S6K expression showed to be negatively correlated with that of miR-486-5p in the NSCLC tissue samples (**Figure S3**), especially in the LUAD samples (**Figures 3H, I**), with their expression being significantly upregulated in most of NSCLC cell lines (**Figures 3N, S**). In accordance with the expression pattern of miR-486-5p in NSCLC tissue samples, both RSK and p70S6K showed to be upregulated in most LUAD tissues, tissues with diameters >= 3 cm and the stage III/IV tissues (**Figures 3J–M, O–R**). Taken together, RSK and p70S6K are confirmed to be targets of miR-486-5p.

**Figure 4:**
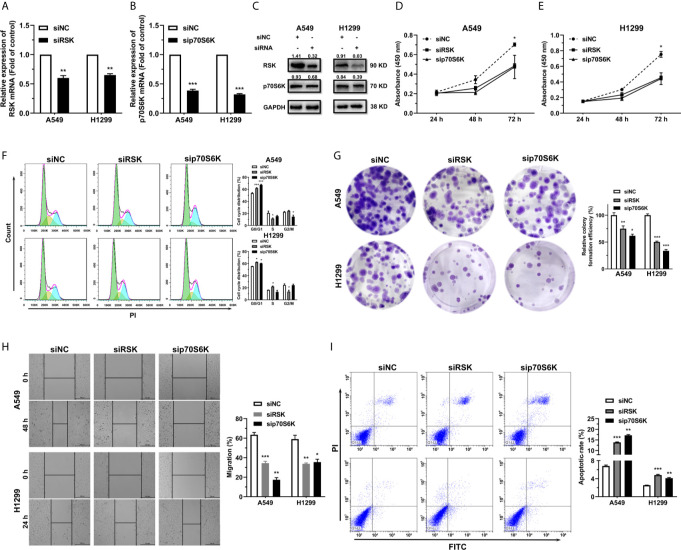
siRSK and sip70S6K suppress NSCLC cell proliferation and promote cell apoptosis. **(A, B)** RNA expression of RSK/p70S6K in siRSK/sip70S6K transfected A549 and H1299 cells compared to siNC transfected ones. **p < 0.01; ***p < 0.001. **(C)** The protein expression of RSK/p70S6K in siRSK/sip70S6K transfected A549 and H1299 cells. **(D–I)** The effect of siRSK/sip70S6K on the proliferation **(D, E)**, cell cycle progress **(F)**, colony formation **(G)**, migration **(H)** and cell apoptosis **(I)** of A549 and H1299 cells. *p < 0.05; **p < 0.01; ***p < 0.001.

To further study the effect of RSK and p70S6K on NSCLC, RSK and p70S6K siRNAs (siRSK 1-3 and sip70S6K 1-3) were transfected into A549 and H1299 cells, resulting in a significant reduction of mRNA (**Figure S4**). As the siRSK -2 and sip70S6K -3 downregulated the mRNA of their respective target most significantly, we chose them for subsequent research (simplified as siRSK and sip70S6K in the main Figures). The siRNAs we chose significantly downregulated the mRNA (**Figures 4A, B**) and protein (**Figure 4C**) expression of RSK and p70S6K. Similar to the effect of the overexpression of miR-486-5p, siRSK and sip70S6K dramatically inhibited the proliferation (**Figures 4D, E**), cell cycle (**Figure 4F**), colony formation (**Figure 4G**) and cell migration (**Figure 4H**) of A549 and H1299 cells, whereas the cell apoptotic rate was upregulated (**Figure 4I**) by the siRNAs. These results showed that the downregulation of RSK and p70S6K could mimic the effects of miR-486-5p in NSCLC cells, indicating that the function of miR-486-5p on NSCLC cell phenotype was exerted at least partly *via* downregulation of RSK and p70S6K.

### Ectopic Express of RSK and p70S6K Rescues the Effect of miR-486-5p

For determination of whether miR-486-5p exerted its function in NSCLC by targeting RSK and p70S6K, we constructed RSK and p70S6K overexpression vectors pcDNA3.1-RSK/pcDNA3.1-p70S6K to find out if restoration of RSK and p70S6K expression could rescue the effects of miR-486-5p.

**Figure 5:**
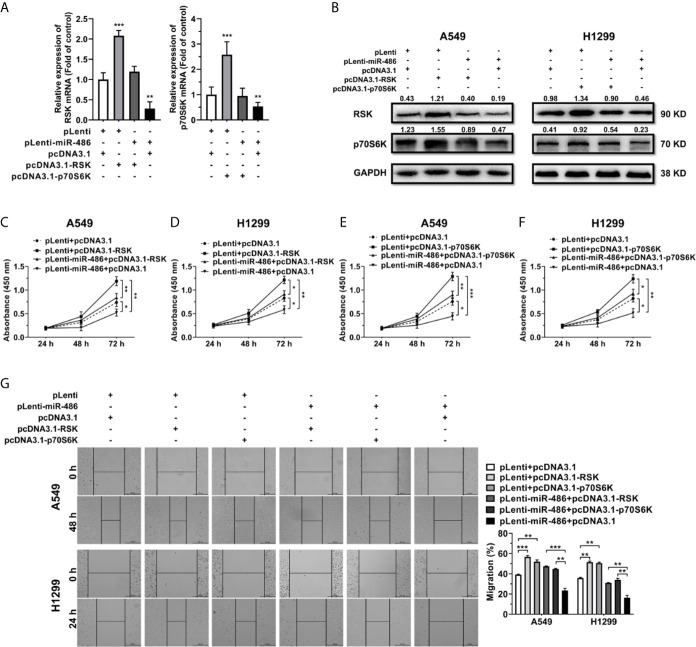
Restoration of RSK/p70S6K rescues the effect miR-486-5p in A549 and H1299 cells. **(A, B)** The relative RNA **(A)** and protein **(B)** expression of RSK/p70S6K in the pLenti/pLenti-miR-486 transfected A549 and H1299 cells cotransfected with pcDNA3.1-RSK/pcDNA3.1-p70S6K or pLenti vectors. **p < 0.01; ***p < 0.001. **(C–G)** The effect of RSK/p70S6K restoration on the proliferation **(C–F)** and migration **(G)** of A549 and H1299 cells. *p < 0.05; **p < 0.01; ***p < 0.001.

Transfection of pcDNA3.1-RSK/-p70S6K in pLenti/pLenti-miR-486 stably transfected A549 and H1299 cells resulted in a marked upregulation of RSK/p70S6K (**Figures 5A, B**). Notably, forced expression of RSK/p70S6K promoted cell proliferation (**Figures 5C–F**) and migration (**Figure 5G**), reversing the inhibition by miR-486-5p. These findings demonstrated that the upregulation of RSK/p70S6K could rescue the effects of miR-486-5p overexpression in NSCLC cells, further verifying the function mechanism of miR-486-5p in NSCLC.

### miR-486-5p Suppresses mTOR Pathway *In Vivo*

**Figure 6:**
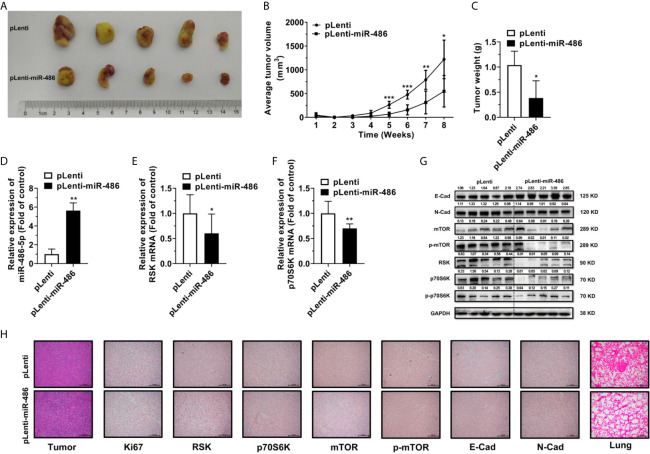
miR-486-5p inhibits tumor growth and metastasis *in vivo*. **(A)** The xenografts images of H1299 cells transfected with pLenti or pLenti-miR-486. **(B)** The growth curve of xenografts from one-week post-injection until sacrifice at week 8. *p < 0.05; **p < 0.01; ***p < 0.001. **(C)** The tumor weight of xenografts at week 8. *p < 0.05. **(D–F)** The RNA expression of miR-486-5p **(D)**, RSK **(E)** and p70S6K **(F)** in pLenti-miR-486 transfected xenografts compared with pLenti transfected ones. *p < 0.05; **p < 0.01. **(G)** The protein expression of E-Cad, N-Cad, mTOR, p-mTOR, RSK, p70S6K and p-p70S6K in xenografts. **(H)** The H&E staining (Left first) and IHC results of Ki67, RSK, p70S6K, mTOR, p-mTOR, E-Cad and N-Cad of the xenografts and the H&E staining (Right first) of the Lung of the tumor-bearing mice.

To explore the effect of miR-486-5p on tumor growth and migration *in vivo*, 5 × 10^6^ H1299 cells expressing either pLenti or pLenti-miR-486 were injected subcutaneously at the left flank and 2.5 × 10^6^ cells intravenously into SCID mice. Upon implantation, tumor volume was measured every week, and mice were sacrificed at week 8. The tumors are displayed in **Figure 6A**. Suppression of tumor growth by miR-486-5p became remarkable at week five and got less significant later (**Figure 6B**). One possible reason is that the tumor growth space is confined as the tumor grows, and nutrients and oxygen are insufficient for the tumor development, ultimately leading to festering. A noted induction in tumor weight was also observed (**Figure 6C**).

Moreover, miR-486-5p expression was significantly upregulated, and RSK and p70S6K were downregulated at the RNA level in the pLenti-miR-486 stably transfected xenografts (**Figures 6D–F**). Protein expression of RSK and p70S6K were also downregulated in the pLenti-miR-486 group, together with a marked reduction on the protein level of phosphorylated p70S6K, as well as the downstream mTOR and phosphorylated mTOR level (**Figure 6G**), indicating the inactivation of the mTOR pathway by miR-486-5p. H&E staining of the tumor tissues was displayed (**Figure 6H**, left panel), and that of the lung specimens showed that miR-486-5p significantly reduced the number and size of metastatic nodes (**Figure 6H**, right panel), indicating miR-486-5p suppressed tumor migration *in vivo*. Immunohistochemistry (IHC) of tumor specimens showed a visible decrease in the expression of Ki67, RSK, p70S6K, mTOR, p-mTOR and N-cadherin (N-Cad), and the E-cadherin (E-Cad) expression was increased compared with control (**Figure 6H**). In accordance with the phenotypic results, the protein expression of E-Cad was significantly upregulated by miR-486-5p, of which the N-Cad showed the opposite results (**Figure 6G**). As E-Cad and N-Cad are two critical cadherins marking the occurrence of the EMT, the IHC results suggested that miR-486-5p suppressed the migration of NSCLC by inhibition of the EMT process. To sum up, these results revealed that miR-486-5p impeded xenograft growth by targeting RSK and p70S6K and further leading to inactivation and inhibition of the mTOR signaling, and the migration of tumor was also hindered partly *via* the inhibition of the EMT process.

## Discussion

The mammalian or mechanistic target of rapamycin (mTOR) is a serine/threonine kinase composed of two protein complexes, mTOR complex 1 (mTORC1) and mTOR complex 2 (mTORC2) that differ in structure and function (29), which has been broadly studied and found to play fundamental roles in cell growth, metabolism and the EMT process (30–32). The overactivation of mTOR is observed in more than 70% of cancers (33, 34), indicating its critical functions in tumorigenesis. RSK and p70S6K are members of RPS6K family (35). Plenty of studies have demonstrated that the mTOR signaling is tightly regulated by miRNAs and long noncoding RNAs (lncRNAs) in various kinds of tumors (36–42), whereas how RSK and p70S6K are regulated is barely understood.

**Figure 7:**
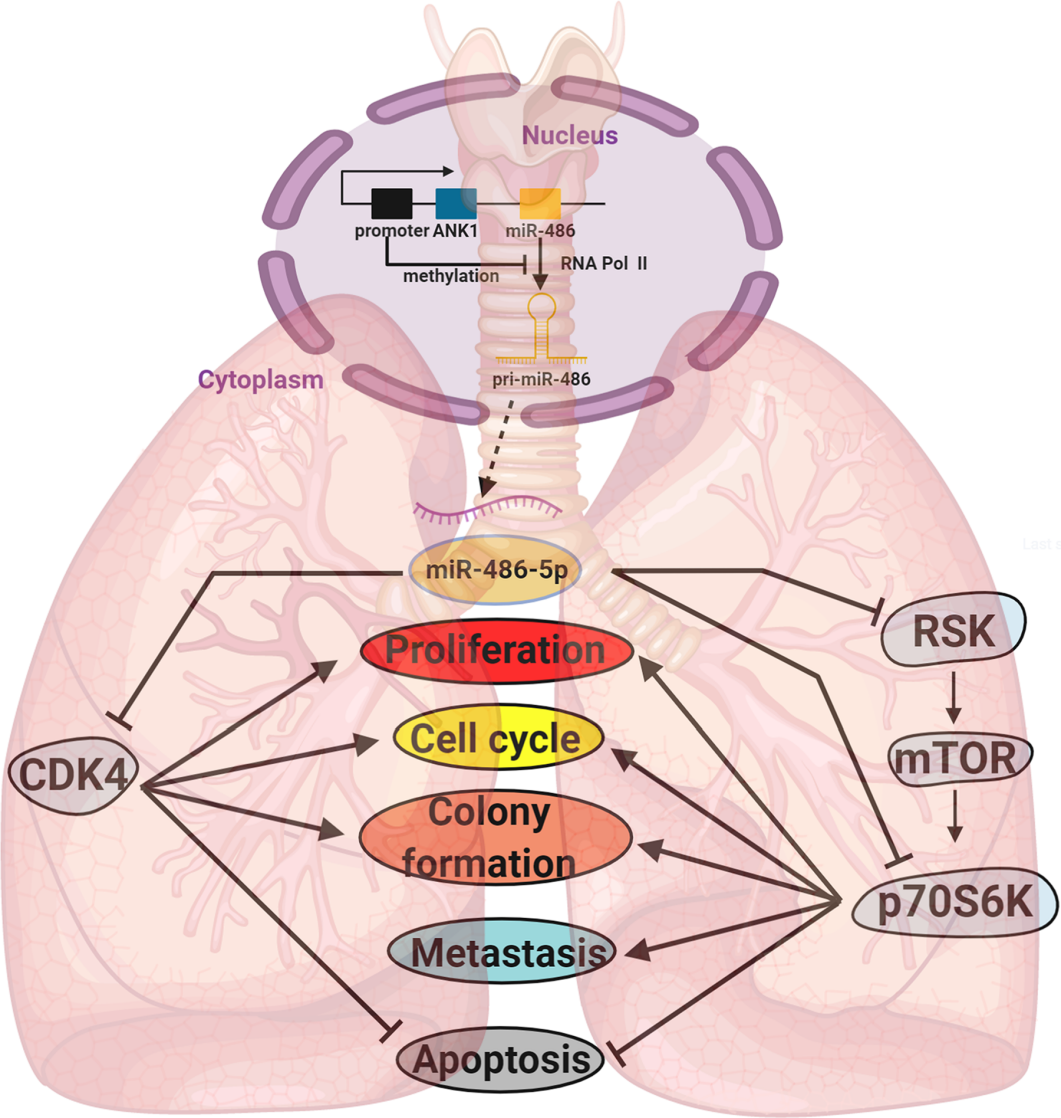
A diagram of the biogenesis and function of miR-486-5p in NSCLC. Locating downstream of *ANK1*, the biogenesis of miR-486-5p is often inhibited by hypermethylation of its promoter. By targeting CDK4, RSK, p70S6K and other genes, miR-486-5p can inhibit NSCLC by hindering cell proliferation and metastasis and promoting cell apoptosis.

Our work revealed that miR-486-5p could hinder lung cancer tumorigenesis through inhibition and inactivation of the mTOR pathway by targeting RSK and p70S6K, and the miR-486-5p/RSK/mTORC1/p70S6K axis (**Figure 7**) could be active.

miR-486-5p is broadly studied in an array of tumor types, including NSCLC (22, 23, 43–45), leukemia (46–48), colorectal cancer (49, 50), renal cell carcinoma (RCC) (51), prostate cancer (52, 53), ovarian cancer (54), hepatocellular carcinomas (55), and breast cancer (56, 57). While most of the studies demonstrated that miR-486-5p played tumor-suppressing function in different malignancies, there are also several studies claiming that it played a tumor-facilitating role in leukemia (46), NSCLC (23), and prostate cancer (52). Thus, we decided that it’s important for us to figure out its function in NSCLC.

Moreover, the downregulation of miR-486-5p in NSCLC is found to be caused by the hypermethylation of the miR-486-5p gene promoter region, which is also confirmed by others’ work (58). To make it clear whether miR-486-5p plays a suppressive or promoting role in NSCLC development, we decided to perform more in-depth research into its function in NSCLC and figure out the underlying mechanisms of this function.

The overexpression of miR-486-5p not only suppressed the expression of RSK and p70S6K both *in vitro* and *in vivo* but also inhibited p-p70S6K, mTOR and p-mTOR. Also, the expression of CDK4 in the pLenti-miR-486 expressing xenografts was significantly downregulated (**Figure S5**), consisting with our former study (22). These findings suggested that miR-486-5p inhibited NSCLC by inactivating of the mTOR signaling.

The expression pattern of miR-486-5p in NSCLC tissue samples has aroused much interest in its function in NSCLC tumorigenesis. Specifically, the miR-486-5p expression is mainly downregulated in the LUAD and early-stage samples, and the expression of RSK and p70S6K showed the reversed tendency. There have been several studies exploring the differentially expressed noncoding RNAs between the LUAD and LUSC patients, thereafter, some lncRNAs, miRNAs, circular RNAs and molecules like interlukin-6R (IL-6R) and IL-1β are considered as diagnostic markers in distinguishing LUAD and LUSC. Our study may establish miR-486-5p as a biomarker for the early diagnosis of NSCLC, especially for the LUAD cases.

## Conclusion

Taken together, our work further confirmed that miR-486-5p acts as a suppressor in NSCLC at least partly by targeting the mTOR signaling and CDK4. With more exploitation of its expression pattern and function mechanism in the *in vivo* model, including exploration of its effective delivery onto the tumor site, we believe it’s a promising target in the clinical treatment of NSCLC.

## Data Availability Statement

The original contributions presented in the study are included in the article/**Supplementary Material**, further inquiries can be directed to the corresponding author/s.

## Ethics Statement

The animal study was reviewed and approved by the institutional Animal Care and Use Committee of Shanghai University. Written informed consent was obtained from the individual(s) for the publication of any potentially identifiable images or data included in this article.

## Author Contributions

YL and CJ designed the study and supervised the experiments. YW and CJ collected the NSCLC specimens and evaluated medical information. LD, WT, HZ and WL carried out the experiments and analyzed the results. LD and YL prepared the manuscript and revised with WT. All authors contributed to the article and approved the submitted version.

## Funding

This study was supported by Pudong New District Plateau Discipline (PWYgy2018-06) and Shanghai Key Laboratory for Molecular Andrology, Shanghai Institute of Biochemistry and Cell Biology, Chinese Academy of Sciences (SLMA-013).

## Conflict of Interest

The authors declare that the research was conducted in the absence of any commercial or financial relationships that could be construed as a potential conflict of interest.
