# Supplementary figures and images for: MicroRNA-486-5p Suppresses Lung Cancer via Downregulating mTOR Signaling In Vitro and In Vivo
Source: Front Oncol. 2021 May 20;11:655236. doi: 10.3389/fonc.2021.655236 (PMC8172781; doi:10.3389/fonc.2021.655236)

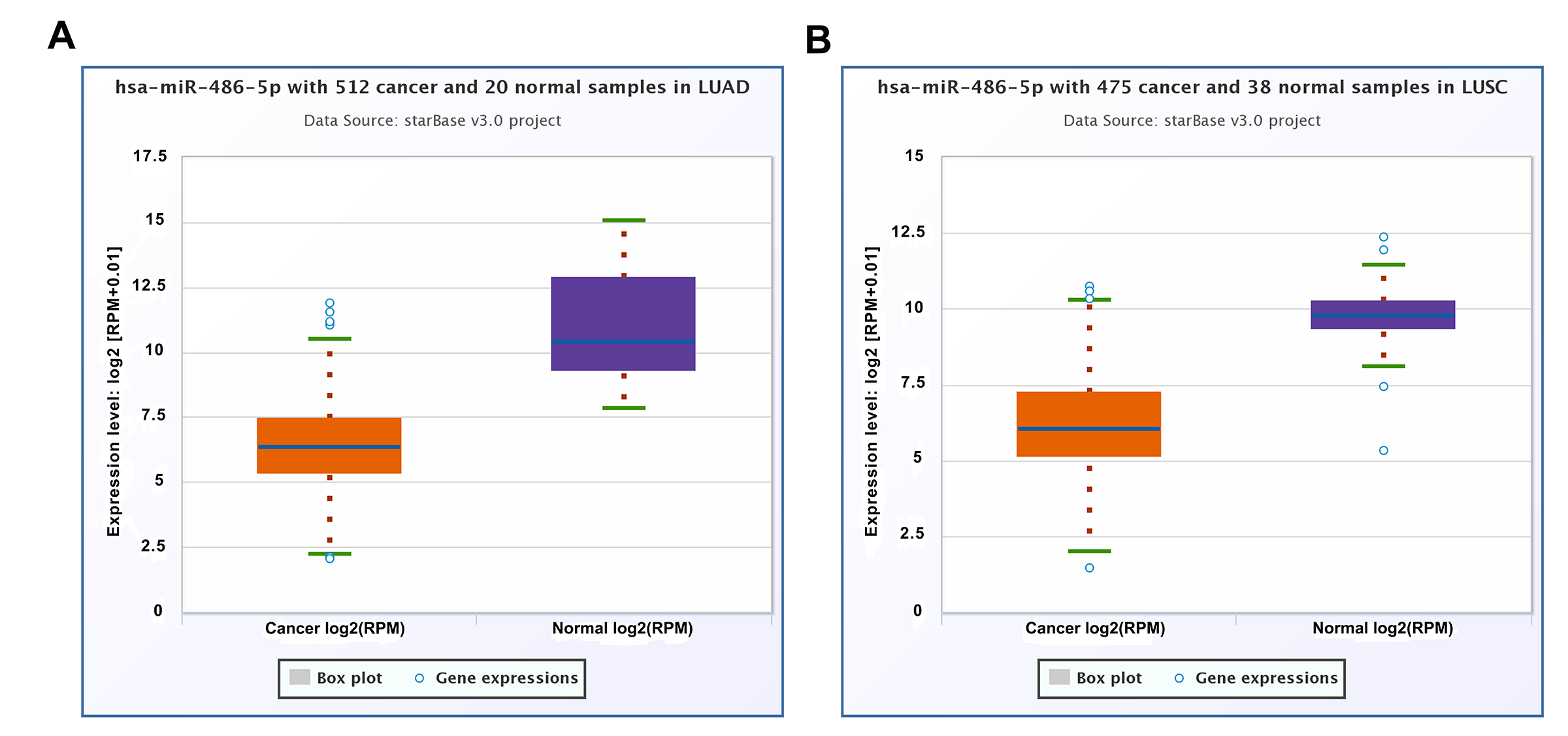

Supplement: Supplementary file 1 [file Image_1.tif]

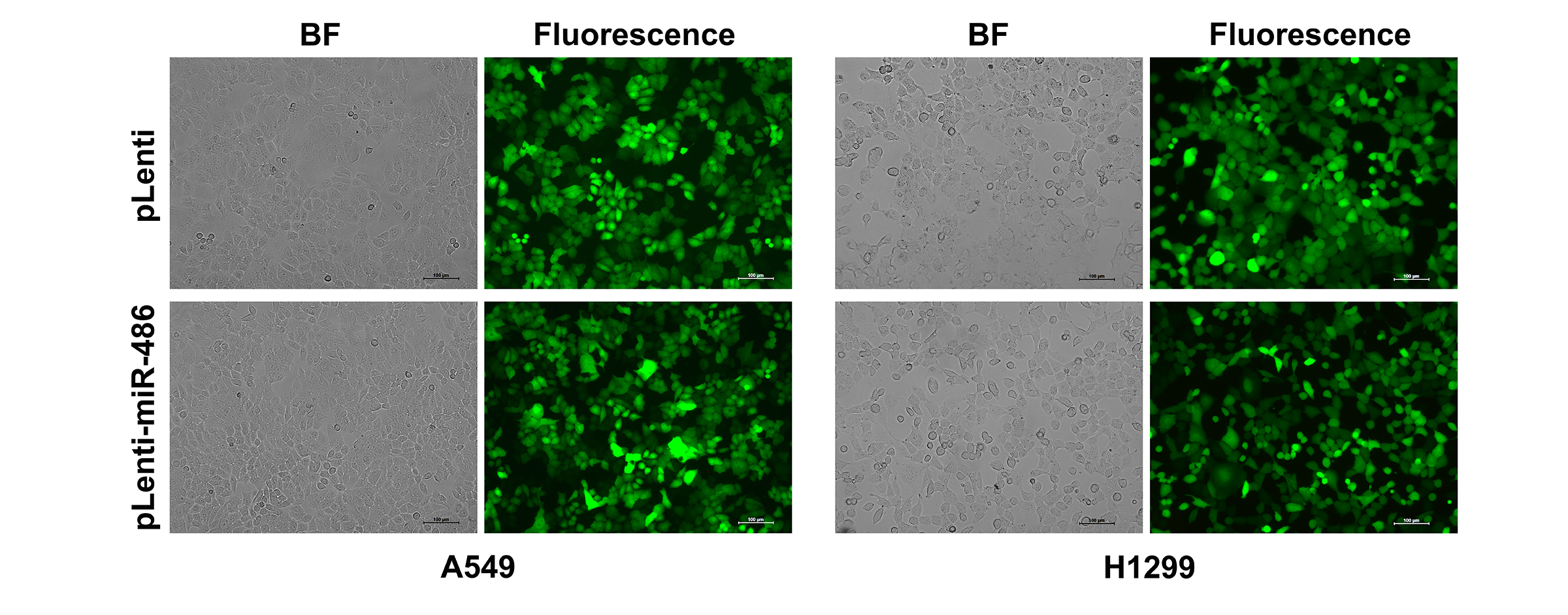

Supplement: Supplementary file 2 [file Image_2.tif]

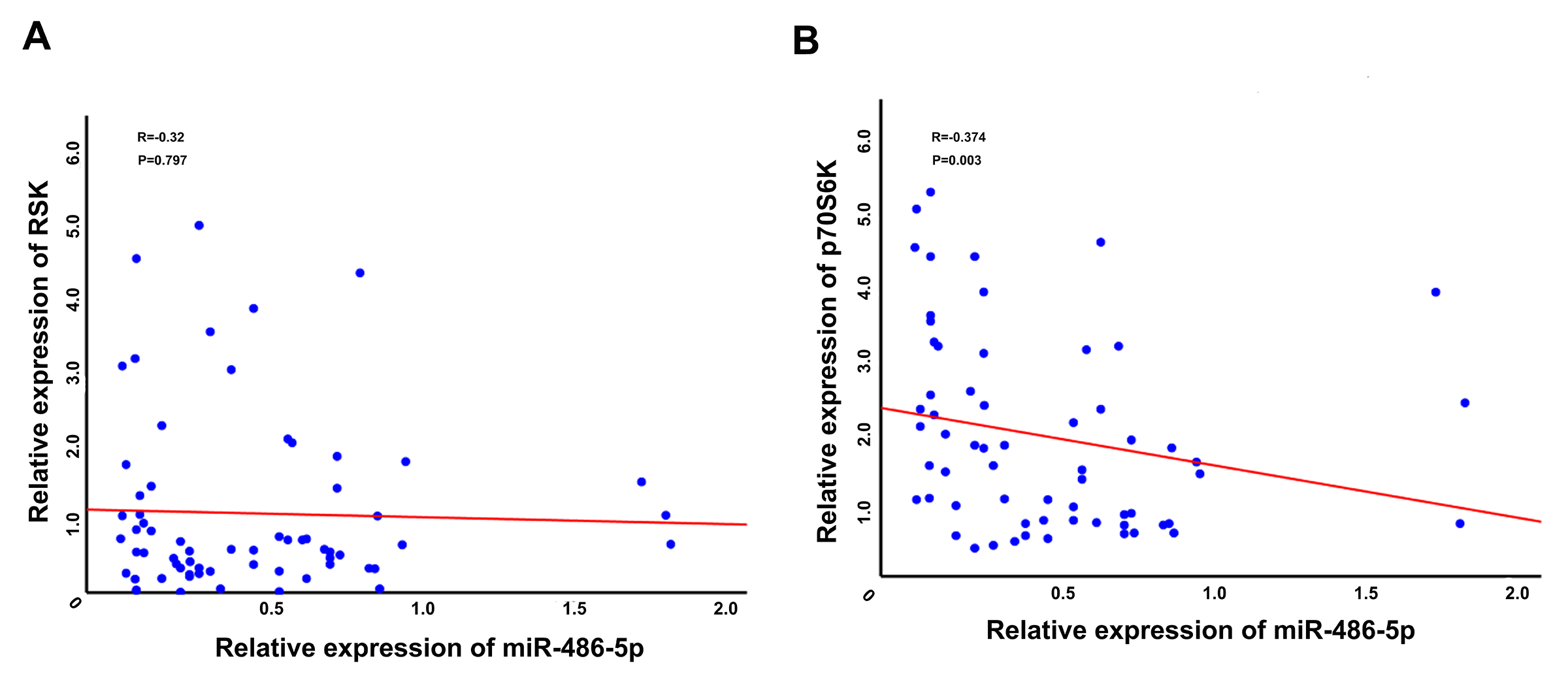

Supplement: Supplementary file 3 [file Image_3.tif]

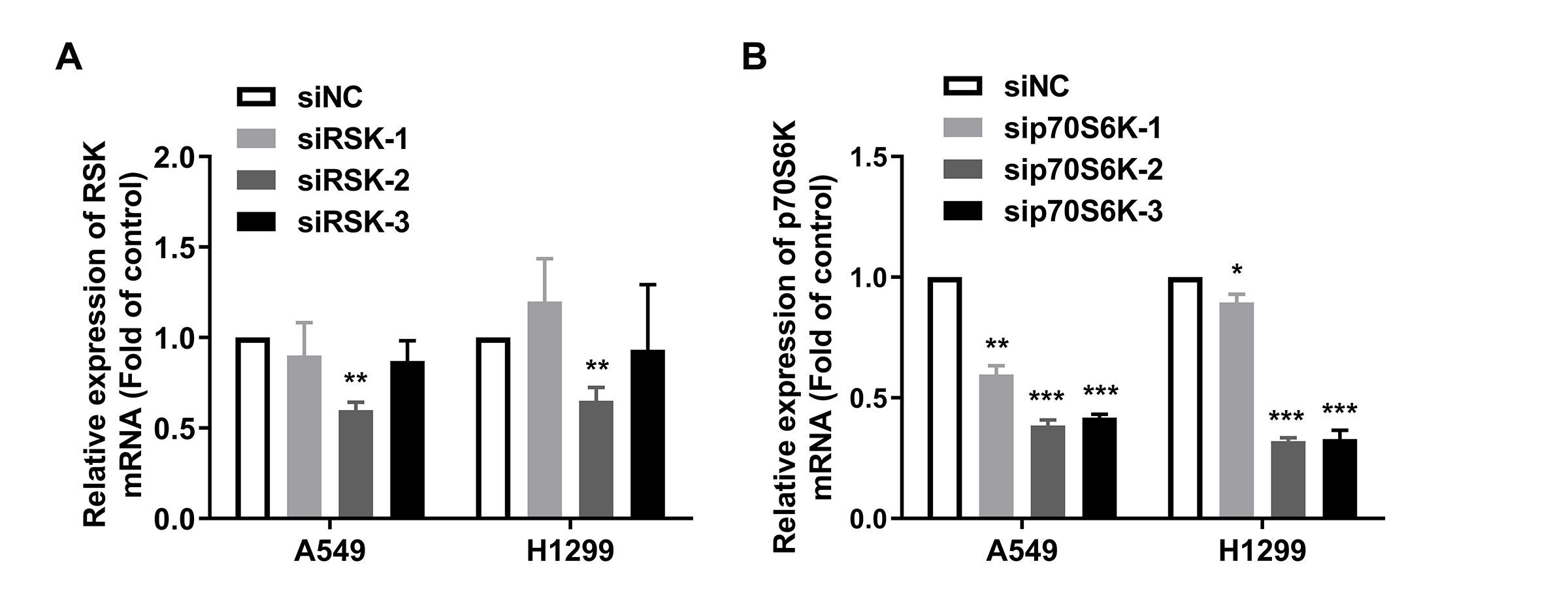

Supplement: Supplementary file 4 [file Image_4.tif]

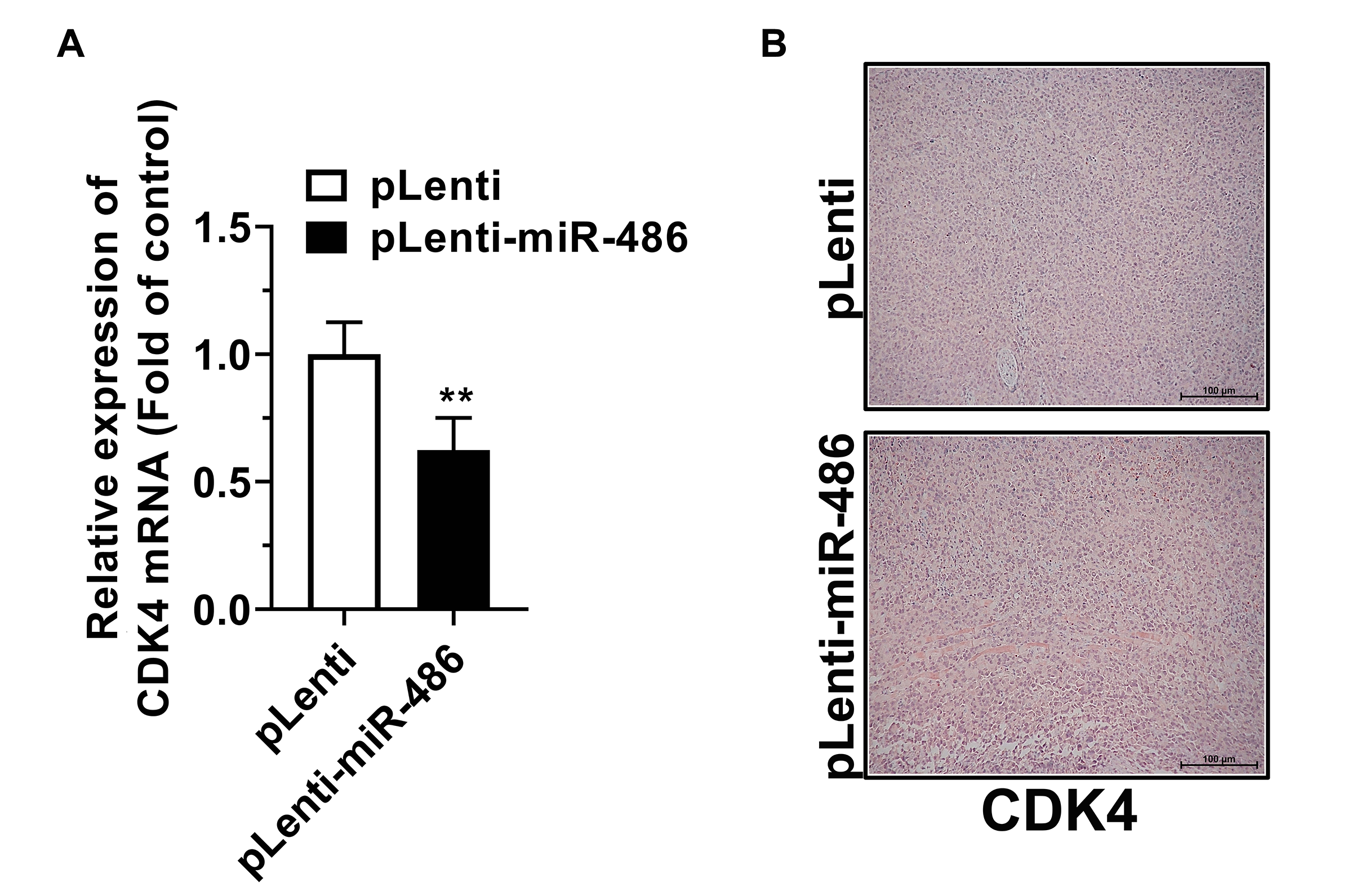

Supplement: Supplementary file 5 [file Image_5.tif]
