# Supplementary material for: MicroRNA-486-5p Suppresses Lung Cancer via Downregulating mTOR Signaling In Vitro and In Vivo
Source: Front Oncol. 2021 May 20;11:655236. doi: 10.3389/fonc.2021.655236 (PMC8172781; doi:10.3389/fonc.2021.655236)
Supplement: Supplementary file 6 [file Table_1.doc]

**Table S1: Information for clinical tissue** samples

| Number | Gender | Tumor stage | Tumor size | Histopathologic classification |
| --- | --- | --- | --- | --- |
| 1 | Male | ⅢA | >3 cm | LUAD |
| 2 | Male | ⅠB | <3 cm | LUAD |
| 3 | Male | ⅠA | <3 cm | LUAD |
| 4 | Male | ⅠA | <3 cm | LUAD |
| 5 | Male | ⅠB | <3 cm | LUSC |
| 6 | Male | ⅠA | <3 cm | LUAD |
| 7 | Female | ⅢA | <3 cm | LUAD |
| 8 | Male | ⅠA | <3 cm | LUSC |
| 9 | Male | ⅠB | <3 cm | LUAD |
| 10 | Male | ⅠB | <3 cm | LUAD |
| 11 | Female | ⅠA | <3 cm | LUAD |
| 12 | Male | ⅡB | >3 cm | LUAD |
| 13 | Male | Ⅳ | >3 cm | LUAD |
| 14 | Female | ⅠB | <3 cm | LUAD |
| 15 | Male | ⅠA | <3 cm | LUAD |
| 16 | Male | ⅠA | <3 cm | LUAD |
| 17 | Female | ⅠA | <3 cm | LUAD |
| 18 | Male | ⅠA | <3 cm | LUSC |
| 19 | Female | ⅢB | >3 cm | LUAD |
| 20 | Male | ⅢA | >3 cm | LUAD |
| 21 | Male | ⅠA | <3 cm | LUAD |
| 22 | Male | ⅠB | <3 cm | LUSC |
| 23 | Female | ⅡB | >3 cm | LUAD |
| 24 | Male | ⅠA | <3 cm | LUAD |
| 25 | Female | ⅢB | >3 cm | LUAD |
| 26 | Male | ⅢA | >3 cm | LUAD |
| 27 | Male | ⅠB | <3 cm | LUAD |
| 28 | Male | ⅡB | >3 cm | LUAD |
| 29 | Male | ⅠA | <3 cm | LUSC |
| 30 | Male | ⅢA | >3 cm | LUSC |
| 31 | Female | ⅡB | >3 cm | LUAD |
| 32 | Female | ⅠA | <3 cm | LUAD |
| 33 | Female | ⅢB | >3 cm | LUAD |
| 34 | Male | ⅠA | <3 cm | LUAD |
| 35 | Male | ⅠA | <3 cm | LUAD |
| 36 | Female | ⅠA | <3 cm | LUAD |
| 37 | Male | ⅠA | <3 cm | LUAD |
| 38 | Male | ⅠA | <3 cm | LUAD |
| 39 | Female | ⅢB | >3 cm | LUAD |
| 40 | Female | ⅡB | >3 cm | LUAD |
| 41 | Male | ⅡB | >3 cm | LUAD |
| 42 | Male | ⅡB | >3 cm | LUAD |
| 43 | Male | ⅠB | <3 cm | LUSC |
| 44 | Male | ⅢA | >3 cm | LUSC |
| 45 | Male | ⅡA | <3 cm | LUAD |
| 46 | Male | ⅢA | >3 cm | LUSC |
| 47 | Female | ⅠA | <3 cm | LUAD |
| 48 | Male | ⅠB | <3 cm | LUSC |
| 49 | Male | ⅠA | <3 cm | LUSC |
| 50 | Male | ⅢA | >3 cm | LUAD |
| 51 | Male | ⅡA | <3 cm | LUAD |
| 52 | Female | ⅢA | >3 cm | LUAD |
| 53 | Male | ⅢA | >3 cm | LUAD |
| 54 | Male | ⅢA | >3 cm | LUSC |
| 55 | Male | ⅠB | <3 cm | LUAD |
| 56 | Male | ⅡA | >3 cm | LUSC |
| 57 | Male | ⅢA | >3 cm | LUAD |
| 58 | Male | ⅠB | <3 cm | LUAD |
| 59 | Male | ⅠB | <3 cm | LUAD |
| 60 | Male | ⅠB | <3 cm | LUAD |
| 61 | Female | ⅠA | <3 cm | LUAD |
| 62 | Male | ⅠB | <3 cm | LUAD |
| 63 | Male | ⅡA | <3 cm | LUSC |
| 64 | Female | ⅢA | <3 cm | LUAD |
| 65 | Male | ⅠB | <3 cm | LUAD |
| 66 | Male | ⅠB | <3 cm | LUAD |
| 67 | Female | ⅢA | >3 cm | LUSC |
| 68 | Female | ⅠB | <3 cm | LUAD |
