# Supplementary material for: MicroRNA-486-5p Suppresses Lung Cancer via Downregulating mTOR Signaling In Vitro and In Vivo
Source: Front Oncol. 2021 May 20;11:655236. doi: 10.3389/fonc.2021.655236 (PMC8172781; doi:10.3389/fonc.2021.655236)
Supplement: Supplementary file 7 [file Table_2.doc]

**Table S2: Sequences used in this study**

| Name | Target mRNA | Sequence (5'-3') |
| --- | --- | --- |
| *hsa*-miR-485-5p probe | miR-485-5p | TCCTGTACTGAGCTGCCCCGAG |
| siRSK (-1) | RSK | GGGATCCTTTGGCAAAGTCTTCC |
| siRSK (-2) | RSK (H1299) | ACGAGAAGAAGGCCTATTCTTTC |
| siRSK (-3) | RSK (A549) | CACCATTGACTGGAATAAGCTAT |
| sip70S6K (-1) | p70S6K | ATGGAACATTGTGAGAAATTTGA |
| sip70S6K (-2) | p70S6K (A549& H1299) | AACATTGTGAGAAATTTGAAATC |
| sip70S6K (-3) | p70S6K | GGCTATGGAAAGGTTTTTCAAGT |
