# Supplementary material for: MicroRNA-486-5p Suppresses Lung Cancer via Downregulating mTOR Signaling In Vitro and In Vivo
Source: Front Oncol. 2021 May 20;11:655236. doi: 10.3389/fonc.2021.655236 (PMC8172781; doi:10.3389/fonc.2021.655236)
Supplement: Supplementary file 8 [file Table_3.doc]

**Table S3: Primer sequences used in this study**

| Name | Usage | Sequence (5'-3') |
| --- | --- | --- |
| 18S RNA | qRT-PCR forward | AGGAATTCCCAGTAAGTGCG |
|  | qRT-PCR reverse | GCCTCACTAAACCATCCAA |
| U6 snRNA | qRT-PCR forward | CTCGCTTCGGCAGCACA |
|  | qRT-PCR reverse | AACGCTTCACGAATTTGCGT |
| RSK | qRT-PCR forward | CAGTGGGCACCTGTATGCTAT |
|  | qRT-PCR reverse | ACGAATGGGTGATTTACATCAGC |
| p70S6K | qRT-PCR forward | CGGGACGGCTTTTACCCAG |
|  | qRT-PCR reverse | TTTCTCACAATGTTCCATGCCA |
| CDK4 | qRT-PCR forward | GGGGACCTAGAGCAACTTACT |
|  | qRT-PCR reverse | CAGCGCAGTCCTTCCAAAT |
| pre-has-miR-486-5p | PCR forward | GCTCTAGAGCAUCCUGUACUGAGC |
|  | PCR reverse | CGGAATTCGTATCCTGTACTGAGC |
| RSK-3'UTR | PCR forward | CCGGAATTCATCTGCACACACCTCCGA |
|  | PCR reverse | AAAACTGCAGAGACCCTGTCCCTCATG |
| p70S6K-3'UTR | PCR forward | GCTCTAGATCCCCCTCCTCCCTTATT |
|  | PCR reverse | CCGGAATTCATCCTCCACCCCTGCCCA |
| RSK-3'mUTR | PCR forward | CTTCCTGCTTCCTGTGGGGTCTCATGTCCTGCTGGCTTCC |
|  | PCR reverse | GACCCCACAGGAAGCAGGAAGTGGTTCTAGAACTCCCAGC |
| p70S6K-3'mUTR-1 | PCR forward | GGTGATATGAATAAGCATTATTTCAGACTGTAAATGGCTT |
|  | PCR reverse | AAATAATGCTTATTCATATCACCATAGCTTTTCGGGTTCT |
| p70S6K-3'mUTR-2 | PCR forward | TCAGCTATTTCTGAATATTTTTTTATTTTATACATGCTGT |
|  | PCR reverse | AAAAAATATTCAGAAATAGCTGATTTTAAAGAAAATAATC |
| RSK-CDS | PCR forward | CGGAATTCATGCCGCTCGCCCAGCTCAAGGAGCCCTGGCCGCTCATGG |
|  | PCR reverse | GCTCTAGACCTGGCCCAGCGGCGAGTGAGGAAGTTGCCATCCACCACCCTGTGA |
| p70S6K-CDS | PCR forward | GCTCTAGAATGAGGCGACGAAGGAGGCGGGACGG |
|  | PCR reverse | CGGAATTCTCATAGATTCATACGCAGGTG |
