# Supplementary material for: MicroRNA-486-5p Suppresses Lung Cancer via Downregulating mTOR Signaling In Vitro and In Vivo
Source: Front Oncol. 2021 May 20;11:655236. doi: 10.3389/fonc.2021.655236 (PMC8172781; doi:10.3389/fonc.2021.655236)
Supplement: Supplementary file 9 [file Table_4.doc]

**Table S4: Information on antibodies used for this study**

| Antibody | Cat. No. | WB | IHC | Specificity | Source |
| --- | --- | --- | --- | --- | --- |
| GAPDH | ab181602 | 1:1000 |  | Rabbit | Abcam |
| Ki67 | ab15580 |  | 1:500 | Rabbit | Abcam |
| CDK4 | ab108357 |  | 1:250 | Rabbit | Abcam |
| RSK | ab32114 | 1:1000 | 1:100 | Rabbit | Abcam |
| p70S6K | #2708 | 1:1000 | 1:480 | Rabbit | CST |
| p-p70S6K | #9208 | 1:1000 | 1:500 | Rabbit | CST |
| mTOR | #2983 | 1:1000 | 1:100 | Rabbit | CST |
| p-mTOR | #5536 | 1:1000 |  | Rabbit | CST |
| p-mTOR | #2976 |  | 1:100 | Rabbit | CST |
| E-Cad | #3195 | 1:1000 | 1:400 | Rabbit | CST |
| N-Cad | #13116 | 1:1000 | 1:125 | Rabbit | CST |
