# Supplementary material for: MicroRNA-486-5p Suppresses Lung Cancer via Downregulating mTOR Signaling In Vitro and In Vivo
Source: Front Oncol. 2021 May 20;11:655236. doi: 10.3389/fonc.2021.655236 (PMC8172781; doi:10.3389/fonc.2021.655236)
Supplement: Supplementary file 10 [file Table_5.doc]

**Table S5: Database and their links in this study**

| Database Name | Links |
| --- | --- |
| miRWalk | <http://mirwalk.umm.uni-heidelberg.de/> |
| TargetScan | <http://www.targetscan.org/vert_72/> |
| TCGA | <https://www.cancer.gov/about-nci/organization/ccg/research/structural-genomics/tcga> |
| miRTarbase | <http://mirtarbase.mbc.nctu.edu.tw/php/index.php> |
| miRBase | <http://www.mirbase.org/> |
| Kaplan-Meier Plotter | <http://kmplot.com/analysis/index.php?p=service&cancer=pancancer_mirna> |
| UALCAN | <http://ualcan.path.uab.edu/> |
